# Survival and Virulence Potential of Drug-Resistant *E. coli* in Simulated Gut Conditions and Antibiotic Challenge

**DOI:** 10.3390/ijerph191912805

**Published:** 2022-10-06

**Authors:** Vankadari Aditya, Akshatha Kotian, Anisha Sanil, Poidal Mohammed-ali Thaseena, Indrani Karunasagar, Vijaya Kumar Deekshit

**Affiliations:** Division of Infectious Diseases, Centre for Science Education and Research, Nitte University, Mangaluru 575018, Karnataka, India

**Keywords:** in vitro gut conditions, ciprofloxacin, transcriptome, antimicrobial resistance, gram-negative pathogens

## Abstract

The gut forms a vital niche for the survival and replication of drug-resistant *E. coli*; however, the role of gut conditions on drug-resistant and sensitive *E. coli* is not clearly understood. The study aims to understand the effect of in vitro gut conditions on the spread of antibiotic resistance among *E. coli* and their ability to adapt to gut conditions. In this study, a multidrug-resistant (J51) and a sensitive (J254) *E. coli* isolate were exposed to a series of in vitro gut conditions and their growth pattern, virulence gene expression and invasion ability were studied. Further, the effect of antibiotic under in vitro gut conditions was also studied. Bile significantly affected the growth of the isolates, and the addition of iron chelator extended the lag phase of the sensitive isolate. Each in vitro gut condition had a differential effect on the expression of virulence genes in both the isolates. Further, the resistant isolate could adhere to and invade Caco2 cell lines better than the sensitive isolate. Most of the downregulated genes showed increased expression upon ciprofloxacin shock under in vitro gut conditions. The transcriptomics study revealed that exposure to bile, led to the downregulation of genes involved in different metabolic pathways. Further downregulation of metabolic pathways on ciprofloxacin shock was also observed. The downregulation of metabolic pathways could be a part of the global response played by the bacteria to adapt to harsh conditions. Reverting these fluctuated pathways could prove to be a novel strategy in combating AMR threat. Overall, bile, in high and low temperature conditions, showed a significant effect on modulating virulence gene expression on the antibiotic challenge. Thus, it is essential to consider the impact of gut conditions on gut pathogens, such as *E. coli*, before prescribing antimicrobial therapy during infection.

## 1. Introduction

Antimicrobial resistance (AMR) is a pandemic of concern that has spread worldwide. It is estimated that 4.95 million deaths in 2019 were associated with AMR bacteria and is further predicted to cause 10 million deaths annually around the globe by 2050 [1]. Among different human pathogens, *E. coli* was the primary cause of death related to AMR in 2019, followed by other pathogens, such as *Staphylococcus aureus*, *Klebsiella pneumoniae*, *Streptococcus pneumoniae*, *Acinetobacter baumannii* and *Pseudomonas aeruginosa* [2]. *E. coli* causes various infections, such as gastroenteritis, urinary tract infections, Gram-negative bacteraemia, etc. The infections caused by *E. coli* depend on factors, such as the host immune system and bacterial virulence. These virulence factors help in the pathogenicity of *E. coli* in evading host immune responses, stimulating inflammatory responses, tissue damage and biofilm formation [3]. Along with these virulence factors, nutrient availability within the host cell is also an essential component for the efficient colonization of *E. coli* [4]. During infection, bacteria must undergo various adaptive changes to withstand stress conditions of the gut, including competition for space and food with the host microbiome, iron availability, bile salt, pH, temperature and NaCl [5,6,7,8]. The majority of these gut environmental conditions are known to have either inhibitory effect or enhance the survival, replication and biofilm formation of *E. coli* [9]. Several antibiotics are used to treat the infections caused by *E. coli*. However, *E. coli* is resistant to different classes of antibiotics, such as *ß*-lactams, fluoroquinolones, aminoglycosides and colistin, which are used to treat infections caused by multi-drug-resistant bacteria [10]. In addition to its multi-drug resistance features, *E. coli* is also known to survive and multiply in varied physiological conditions within the host [10]. Many reports have studied the effect of gut physiological conditions and antibiotics individually on the transcriptomic profile of *E. coli* [11,12]. However, the difference in the growth, virulence, survival, conjugation ability, transcriptome profiling, invasion and replication of drug-resistant and sensitive *E. coli* in simulated in vitro gut conditions and in the presence of sub-lethal concentrations of antibiotics is not clearly understood. The effect of antibiotics at different concentrations varies between different microorganisms and it might also get influenced by the presence of in vitro gut conditions. Further, the sub-lethal concentration of ceftriaxone showed varied expression of OMPs in *Salmonella* Typhi [13]. The sub-lethal concentrations of antibiotics, such as trimethoprim, quinolones, furazolidone, etc., are known to promote the production of Shiga toxins in EHEC-O157 [14,15,16]. Hence, this study attempts to elucidate the role of in vitro gut conditions and antibiotics on the virulence potential of drug-resistant and sensitive *E. coli* isolated from clinical sources. 

## 2. Materials and Methods

### 2.1. Bacterial Strains and Growth Conditions

A total of 80 *E. coli* strains, previously isolated from clinical sources and stored in institutional repository, were used in the study. Isolates were revived using Luria–Bertani (LB) broth (HiMedia Laboratories Pvt. Ltd., Maharashtra, India) and incubated at 37 °C overnight in a shaker incubator. The isolates were also screened for virulence genes and plasmids. 

### 2.2. Screening of Virulence Genes

All 80 isolates were screened for the presence of virulence genes by PCR. Briefly, PCR was performed in a 30 μL reaction consisting of 22.1 μL of ultra-pure water, 3 μL of assay buffer (10X) (100 mM Tris-HCL (pH 9), 1.5 mM MgCl_2_, 50 mM KCL and 1% gelatin), 0.6 μL of four dNTP mix (200 mM), forward and backward primers (10 pmol of each) (Table S1), *Taq* DNA polymerase (1.0 U) and 2 μL of template DNA in a thermal cycler (Bio-Rad, USA). 

### 2.3. Extraction and Profiling of Plasmid DNA

Extraction of plasmid DNA was performed using the alkaline lysis method [17]. The extracted DNA was electrophoresed at 90 V for 1 h and visualized under UV-trans-illuminator. Plasmid profiles were generated by grouping the strains on the basis of the number of bands obtained and their molecular size [18].

### 2.4. Conjugation Experiment

Quinolone- and fluoroquinolone-resistant isolates were screened for the presence of plasmid-mediated quinolone resistance genes. The isolates that are positive for *qnr* genes were tested for their conjugation ability in LB broth using *E. coli* J53AzR as the recipient strain. Briefly, 0.5 mL of both donor and recipient cultures from the mid-logarithmic phase of growth were added to 4 mL of fresh LB broth and incubated overnight under static conditions. The overnight grown cultures were then plated on Tryptic soy agar (TSA) plates supplemented with sodium azide (300 mcg/mL) (HiMedia Laboratories Pvt. Ltd., Maharashtra, India) and ciprofloxacin (1 mcg/mL) (HiMedia Laboratories Pvt. Ltd., Maharashtra, India) to select the transconjugants [19].

### 2.5. Growth Kinetics of MDR and Sensitive E. coli under In-Vitro Gut Conditions

Based on the antibiogram obtained in our previous study by Aditya et al. [9], an isolate showing least susceptibility (J51) and an isolate showing maximum susceptibility (J254) to the tested antibiotics, such as ampicillin (10 µg), cefotaxime (30 µg), imipenem (10 µg), ciprofloxacin (5 µg), nalidixic acid (30 µg), levofloxacin (5 µg), tetracycline (30 µg), nitrofurantoin (300 µg) and co-trimoxazole (25 µg), were selected to study the effect of in vitro gut conditions on growth kinetics, virulence gene expression, adhesion and invasion.

In order to check the effect of in vitro gut conditions on growth, the isolates J51 and J254 were grown separately in LB broth supplemented with 3% Sodium cholate (Bile condition) (Sigma-Aldrich, St. Louis, MO, USA), 0.3 M NaCl (Osmotic) (HiMedia Laboratories Pvt. Ltd., Maharashtra, India), 0.2 mM 2,2-dipyridyl (Low iron (LI)) (Sigma-Aldrich), 100 µM Ferric chloride (FeCl_3_) (High iron (HI)) (HiMedia Laboratories Pvt. Ltd., Maharashtra, India), acidic condition (pH 5.8), high temperature (HT) (39 °C) and low temperature (LT) (25 °C). Bacteria grown in normal conditions were used as a control. Briefly, overnight grown cultures were used as inoculum for each in vitro gut condition. The flasks with each culture growing along with individual in vitro gut conditions were incubated at 37 °C in a shaker incubator at 200 rpm (New Brunswick, Eppendorf, Enfield, CT, USA). The optical density was measured at 600 nm (OD_600_) for every two hours up to 30 h. For bile condition, 100 µL of the culture was plated onto LB agar and the viable colonies were counted after overnight incubation at 37 °C and expressed as log (OD_600_ × 1000). All the experiments were performed in triplicates.

### 2.6. Determination of Minimum Inhibitory Concentration (MIC) on Exposure to In-Vitro Gut Conditions

MIC was determined to check the effect of infection-related in vitro gut conditions, such as bile, osmotic, high and low iron, pH and temperature conditions, on MIC variation in resistant and sensitive isolate by using Ezy MIC™ strips of ampicillin, co-trimoxazole, imipenem, nalidixic acid, ciprofloxacin, tetracycline, nitrofurantoin and chloramphenicol (HiMedia Laboratories Pvt. Ltd., Maharashtra, India). Both the isolates were grown up to mid-exponential phase (MEP) under in vitro gut conditions and swabbed onto Muller Hinton agar (MHA) plates. MIC strips were placed onto plates using an applicator followed by incubation at 37 °C for 16–18 h. The result was read where the ellipse intersects the MIC scale on the strip for the tested antibiotics.

### 2.7. Effect of Simulated Gut Conditions and Ciprofloxacin Shock on the Expression of Different Virulence Genes

The expression of virulence genes under different infection-related in vitro gut conditions was studied for both the *E. coli* isolates using qPCR. The isolates were grown up to MEP under individual in vitro gut conditions and subjected to ciprofloxacin shock for 30 min and aliquots were collected for RNA isolation to check the expression of virulence genes. The antibiotic concentration was selected based on the CLSI (2018) breakpoint. The RNA extraction was done using the TRIzol method. DNase treatment was done as per manufactures protocol (Thermo Fisher Scientific Inc, USA). The cDNA synthesis was carried out using TaKaRa PrimeScript^TM^ First Strand cDNA synthesis kit. The cDNA that was synthesized was used as the template for qPCR. To rule out random amplification, melt curve analysis was performed and 2^−ΔΔct^ formula was used to quantify mRNA transcripts. A 16S rRNA was used as an internal control. The qPCR was carried out in a 10 µL reaction volume containing 5 µL of 2X universal SYBR green master mix, 200 nm of forward and reverse primers and 1 µL of cDNA (100 ng/µL) and molecular-grade water to adjust the volume. The qPCR was performed using QuantStudio^TM^ 5 Real-Time PCR detection system (Applied Biosystems, USA). The cycling conditions are as follows: initial denaturation at 95 °C for 3 min, followed by 40 cycles of denaturation at 95 °C for 10 s, annealing at 52 °C for 20 s and final extension at 72 °C for 20 s. Data acquisition at the end of each elongation step was carried out using QuantStudio ^TM^ 5 software.

### 2.8. Bacterial Adhesion and Invasion Assay 

The Caco-2 (RRID: CVCL_0025) cell lines were procured from National Centre for Cell Science (NCCS), Pune, India and passaged and preserved at –196 °C. The cell lines were revived and plated onto 12-well cell culture plates (Eppendorf, Germany) with a density of 10^5^ cells/well and incubated at 37 °C with 5% CO_2_ for 24 h. A monolayer of cells showing 70–80% confluence was selected for further experiment. The plates containing cells were washed with Dulbecco’s phosphate-buffered saline (D-PBS). The pre-existing medium was removed, and then added with Dulbecco’s Modified Eagle Medium (DMEM), containing 10% fetal bovine serum (FBS) w/o antibiotics prior to bacterial infection. Bacteria cells grown under different in vitro gut conditions were used to infect Caco-2 cells with a multiplicity of infection (MOI) of 10 and incubated with 5% CO_2_ in an incubator. For adhesion assay, after 30 min of incubation, the monolayers were washed twice with D-PBS and lysed using 500 μL of cold 0.1% Triton X-100. The bacteria were enumerated by plating the serially diluted aliquots and grown overnight at 37 °C [20]. For invasion assays, the monolayers incubated with bacteria for one and half hours were washed with D-PBS and incubated further for 30 min with DMEM and 10% FBS-containing gentamicin (100 µg/mL) to kill extracellular bacteria. Caco-2 monolayers were washed and lysed, and intracellular bacteria were enumerated, as mentioned above.

### 2.9. Transcriptome Analysis of MDR Strain Grown in 3% Bile

#### 2.9.1. Bile Exposure

For transcriptome analysis, the MDR isolate J51 was grown in 3% bile and after reaching 0.6 O. D, the bacterial pellet was collected for RNA isolation. Similarly, to study the effect of antibiotic shock, 1 µg/mL of ciprofloxacin was added to the culture at MEP (0.6 O.D). The culture was further allowed to grow for 30 min and pellets were collected for RNA isolation.

#### 2.9.2. Total RNA Isolation, cDNA Library Construction and Analysis

Total RNA isolation, cDNA synthesis and sequencing were carried out at Biokart India Pvt. Ltd., Bengaluru, India. Briefly, total RNA was extracted from the control, bile (B) grown culture and from the culture grown in bile, exposed to antibiotic shock (BAB) for 30 min by using the TRIzol method. DNase treatment was done as per the manufacturer’s protocol using NEB (M0303L) kit. PCR was performed to confirm that the genomic DNA was removed by treatment with DNase I. Total RNA concentration and quality were measured using Qubit. Prior to sequencing, ribosomal RNA (rRNA) was removed using NEB (E7850X) kit. The quality of the RNA was analysed by checking the RNA integrity number (RIN) using Bioanalyzer (Agilent Technologies Pvt Ltd., Santa Clara, CA, USA). Samples with RIN values ≥10 were subjected to library preparation. RNA-Seq libraries were prepared by NEB (E7770L) mRNA kit. After validation, libraries were quantified using Bioanalyzer. Sequencing was performed using Illumina 150 bp PE platform. Raw reads were subjected to a quality check using FastQC and MultiQC. Adapters were removed from the raw reads using Trim Galore. A Phred score of >30 was obtained for all the samples. The Rockhopper tool was used to identify the adaptors as well as the transcripts. The reads were mapped against *E. coli* K-12 MG1655 strain reference genome using the software Rockhopper.

#### 2.9.3. Gene Ontology and Cluster Analysis

DAVID (database for annotation, visualization and integrated discovery) (https://david-d.ncifcrf.gov accessed on 11 August 2022), an online tool, was used to determine the biological processes of gene ontology for the differentially expressed genes [21]. The functional annotation clustering and Kyoto encyclopedia of genes and genomes (KEGG) pathways were obtained within the analysis of DAVID [22]. The enrichment score (ES) threshold was fixed to 0.5.

#### 2.9.4. Transcriptome Validation by qPCR

For transcriptome validation, two upregulated genes—namely *csgB*, responsible for curli production, and multiple antibiotic resistance genes, *marB*—and two downregulated genes related to motility *fliC* and *fimA* were selected. The qPCR was carried out, as described earlier. 

#### 2.9.5. Validation of Transcriptome Data by Motility Test

The motility of MDR isolate J51 was analysed on exposure to bile and combination of bile and ciprofloxacin by growing them in soft agar medium. Briefy, J51 grown in bile, control condition (devoid of bile) and combination of bile and ciprofloxacin was inoculated to soft agar containing 1% tryptone, 0.5% NaCl and 0.5% agar (wt/vol). Inoculation of these cultures was done using sterile straight needle by stabbing through the centre of the semisolid agar tubes approximately half the depth of the medium. The tubes were incubated at 37 C for 16–18 h and observed for the growth. The motility was observed based on the difused growth produced by the strain. 

#### 2.9.6. Statistical Analysis

Growth kinetics, adhesion and invasion ability of MDR (J51) and sensitive strain (J254) under in vitro gut conditions were performed in triplicates. Statistical significance was determined by using unpaired *t*-test (*p* < 0.05).

## 3. Results

### 3.1. Screening of Virulence Genes–Occurrence of traT Gene in Majority of the Isolates

Among the virulence genes screened, 60% (48) of the isolates carried the *traT* gene that encodes for serum resistance, while 58% (46) harboured *ompT* gene that encodes outer membrane proteases. The alpha-hemolysin toxin-encoding gene *hlyA* was prevalent in 28% (22) and *cnf-1*, the cytotoxic necrosis factor-1 was present in 15% (12) of the isolates, respectively. The gene encoding invasion protein *ibeA* and O antigen polymerase *rfc* was absent in all the tested isolates. The *traB* related to pilin that forms conjugative pilli was present in 35% (28) of the isolates. However, none of the isolates harbored *traA* gene.

### 3.2. Presence of Multiple Plasmids in MDR Strains 

A total of 80 isolates were screened for their plasmid profiling. The number of plasmids varied in isolates from one to four with the size range of 0.8 to >33.5 kb. A total of 4% (3) of the isolates carried four plasmids and 11% (9) of the isolates carried three plasmids, 14% (11) carried two plasmids and 51% (41) of isolates carried single plasmid, while 20% (16) of the isolates did not carry any plasmid.

### 3.3. Trans-Conjugation Ablity of Quinolone Resitant Isolates

Out of 79 quinolone-resistant isolates, 2 isolates were positive for *qnrA*, 6 were positive for *qnrB*, and 8 isolates were positive for *qnrS.* All the *qnr*-positive isolates were tested for their conjugation ability to transfer quinolone resistance from donor to recipient. However, no trans-conjugants were obtained. 

### 3.4. Reduction in Growth Kinetics of MDR and Sensitive E. coli upon Bile Challenge 

**Figure 1:**
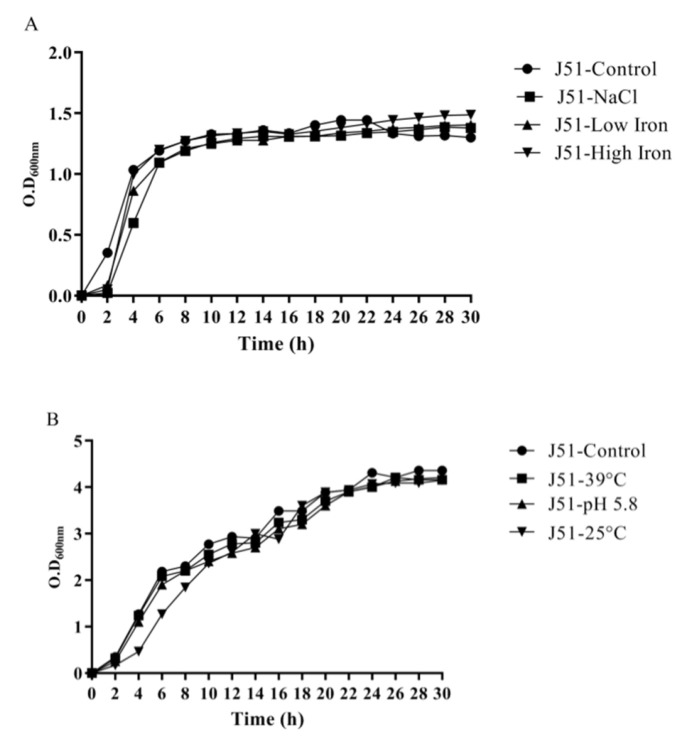

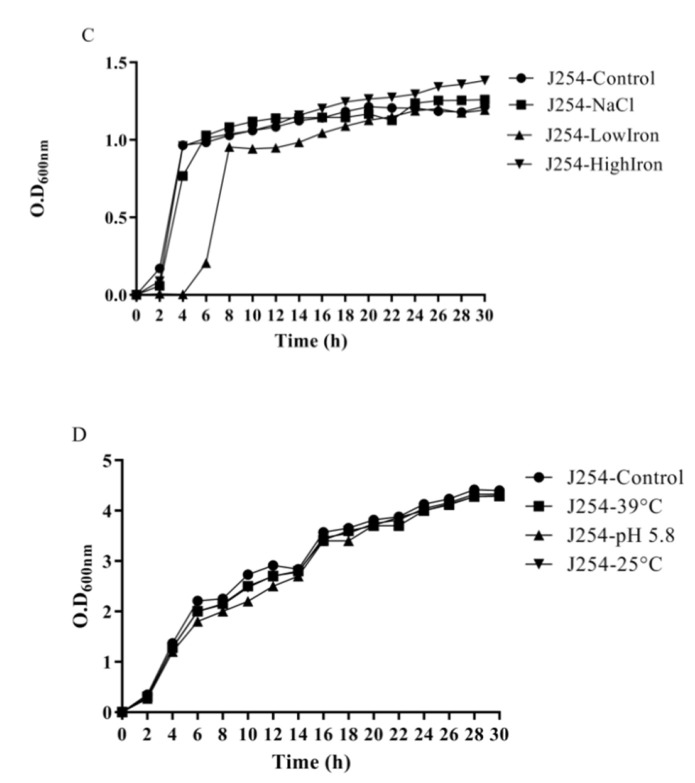
Growth kinetics of MDR *E. coli* J51 (**A** and **B**) and J254 (**C** and **D**) under control and in vitro gut conditions. Unpaired *t*-test was performed and *p* < 0.05 was considered significant. (**A**) Effect of NaCl, low and high iron on growth kinetics of MDR isolate (J51), (**B**) Effect of high temperature, pH, low temperature on growth kinetics of MDR isolate (J51), (**C**)Effect of NaCl, low and high iron on growth kinetics of sensitive isolate (J254), (**D**) Effect of high temperature, pH, low temperature on growth kinetics of sensitive isolate (J254).

**Figure 2:**
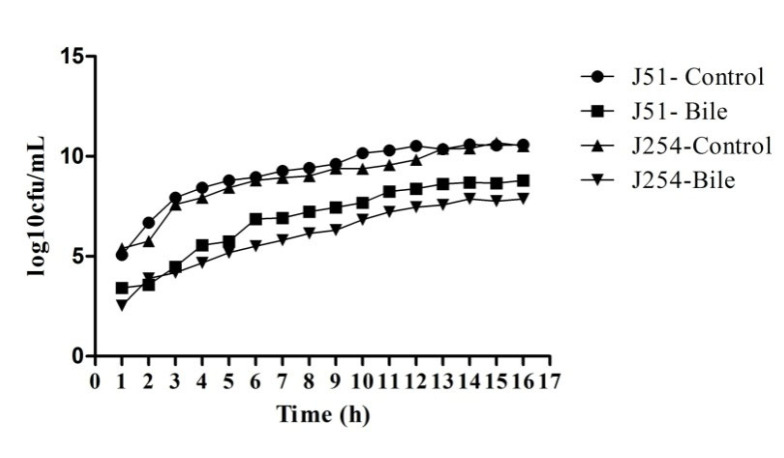
Growth kinetics of the isolates (J51 and J254) under control and bile (3%) conditions. Unpaired *t*-test was performed and *p* < 0.05 was considered significant.

Both the isolates (J51 and J254) were able to withstand the gut physiological conditions that are tested. Unpaired *t*-test (*p* < 0.05) revealed no significant difference between MDR (J51) and sensitive strain (J254) in all the tested in vitro conditions except for bile. However, under LI condition the quinolone-sensitive isolate showed a prolonged lag phase, when compared to the resistant isolate. No potential advantage was observed in the growth by the addition of ferric chloride. A reduction in cell density was observed in both the isolates when exposed to bile, indicating that bile has a considerable effect on the growth patterns of isolates (Figure 1 and Figure 2).

### 3.5. Variation in Minimum Inhibitory Concentration (MIC) on Exposure to In-Vitro Gut Conditions

The majority of the in vitro gut conditions did not have any effect on MIC levels for tested antibiotics in both the isolates. A slight variation in MIC level (from 8 µg/mL to 12 µg/mL) for nitrofurantoin was observed in J51 for in vitro gut conditions, such as HI, bile, and NaCl, and in J254 for HI and bile. Similarly, variation in MIC level (from 0.094 µg/ mL to 0.125 µg/mL) for ciprofloxacin was observed in J254 for HI, bile and LT conditions (Table S2 and S3).

### 3.6. Effect of In-Vitro Gut Conditions and Ciprofloxacin Shock on the Expression of Virulence Genes

**Figure 3:**
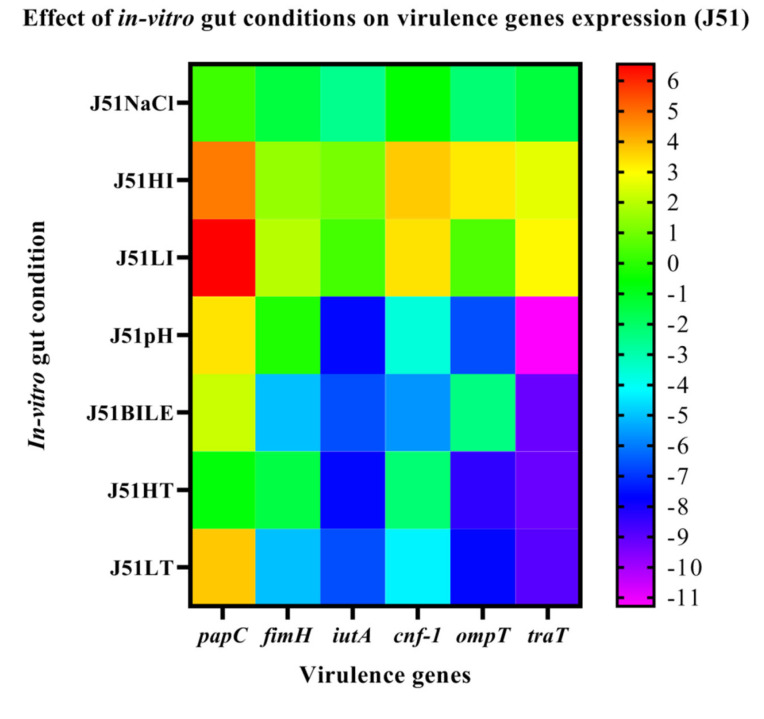
The expression level of virulence genes on exposure to in vitro gut conditions in J51 are presented using fold change values transformed to log2 format. The color scale indicating log2 (fold-change values) is shown towards the right of the heat map.

**Figure 4:**
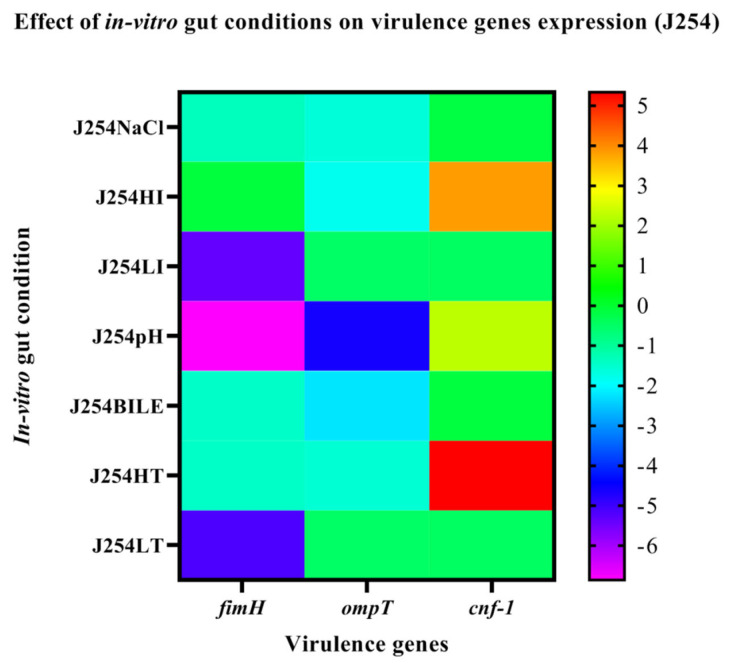
The expression level of virulence genes on exposure to in vitro gut conditions in J254 are presented using fold change values transformed to log2 format. The color scale indicating log2 (fold-change values) is shown towards the right of the heat map.

**Figure 5:**
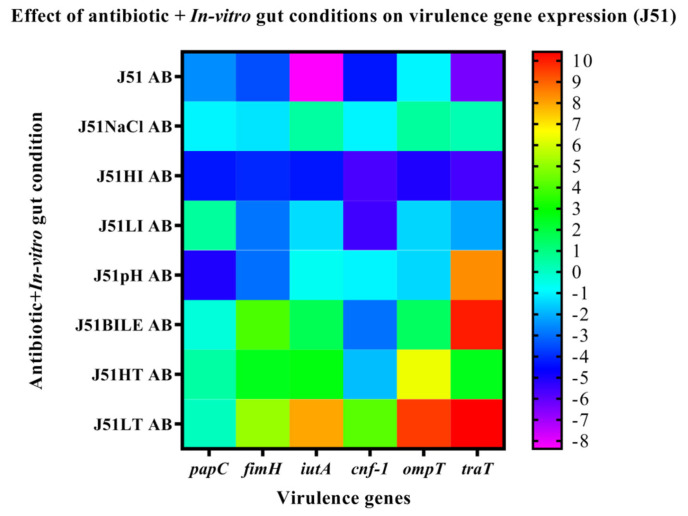
The expression level of virulence genes on exposure to antibiotic + in vitro gut conditions in J51 are presented using fold change values transformed to log2 format. The color scale indicating log2 (fold-change values) is shown towards the right of the heat map.

**Figure 6:**
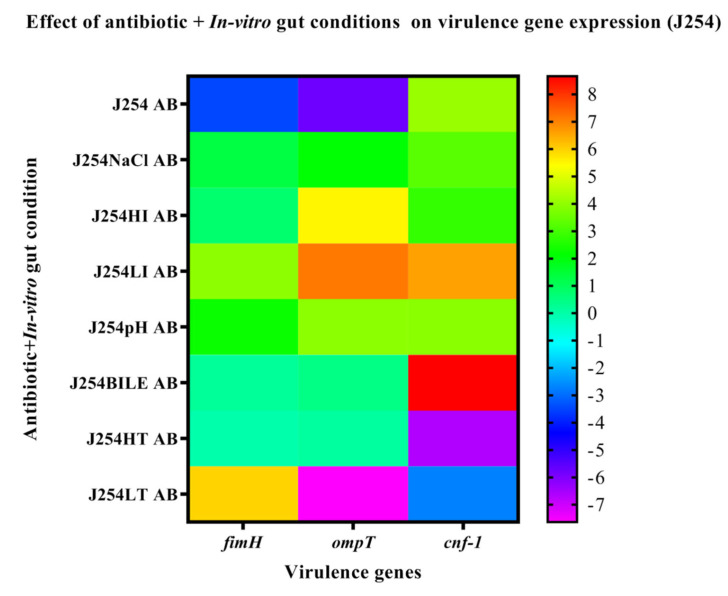
The expression level of virulence genes on exposure to antibiotic + in vitro gut conditions in J254 are presented using fold change values transformed to log2 format. The color scale, indicating log2 (fold-change values), is shown towards the right of the heat map.

The MDR isolate J51 was positive for the virulence genes, such as *papC, fimH, iutA, cnf-1, ompT* and *traT*, while the sensitive isolate harbored only *fimH, ompT* and *cnf-1* genes. The expression of genes on exposure to in vitro gut conditions revealed upregulation of *papC* in J51 under all the tested conditions except HT. The genes *iutA, cnf-1, ompT, traT, fimH* were downregulated in J51 under all the tested conditions except in HI and LI (Figure 3). However, in J254 the *fimH* and *ompT* genes were downregulated under all the conditions, while *cnf-1* gene was upregulated under HI, pH and HT (Figure 4). On antibiotic challenge in J51, all the genes were downregulated under LI and HI condition and were upregulated under LT and in HT. Similarly, under bile conditions, all the genes were upregulated except for *cnf-1* (Figure 5). In J254, all the tested genes were upregulated under NaCl, HI and LI, pH and bile conditions, except under HT and LT on the antibiotic challenge (Figure 6).

### 3.7. Adhesion and Invasion Potential of MDR and Sensitive Isolate

**Figure 7:**
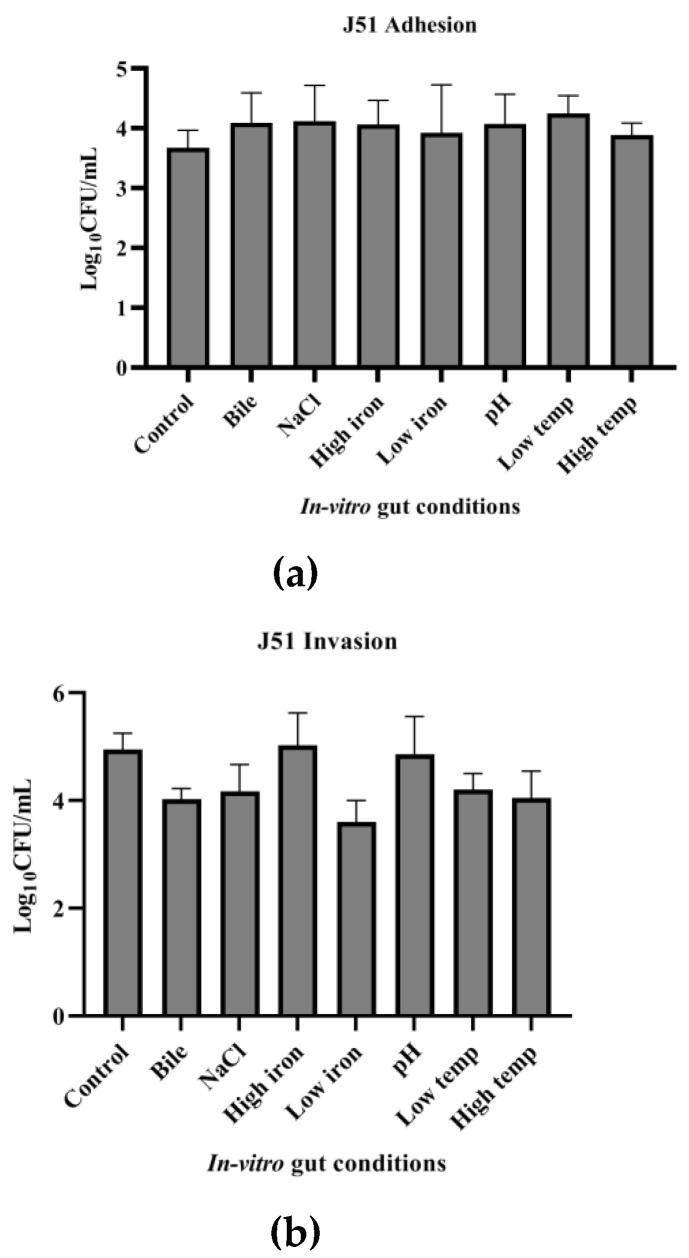
Effect of in vitro gut conditions on adhesion and invasion ability of MDR isolate J51 to Caco-2 cell lines. Error bars represent standard deviations from triplicates. Unpaired *t*-test was performed and *p* < 0.05 was considered significant. (**a**) Effect of in vitro gut conditions on adhesion ability of MDR isolate (J51), (**b**) Effect of in vitro gut conditions on invasion ability of MDR isolate (J51).

**Figure 8:**
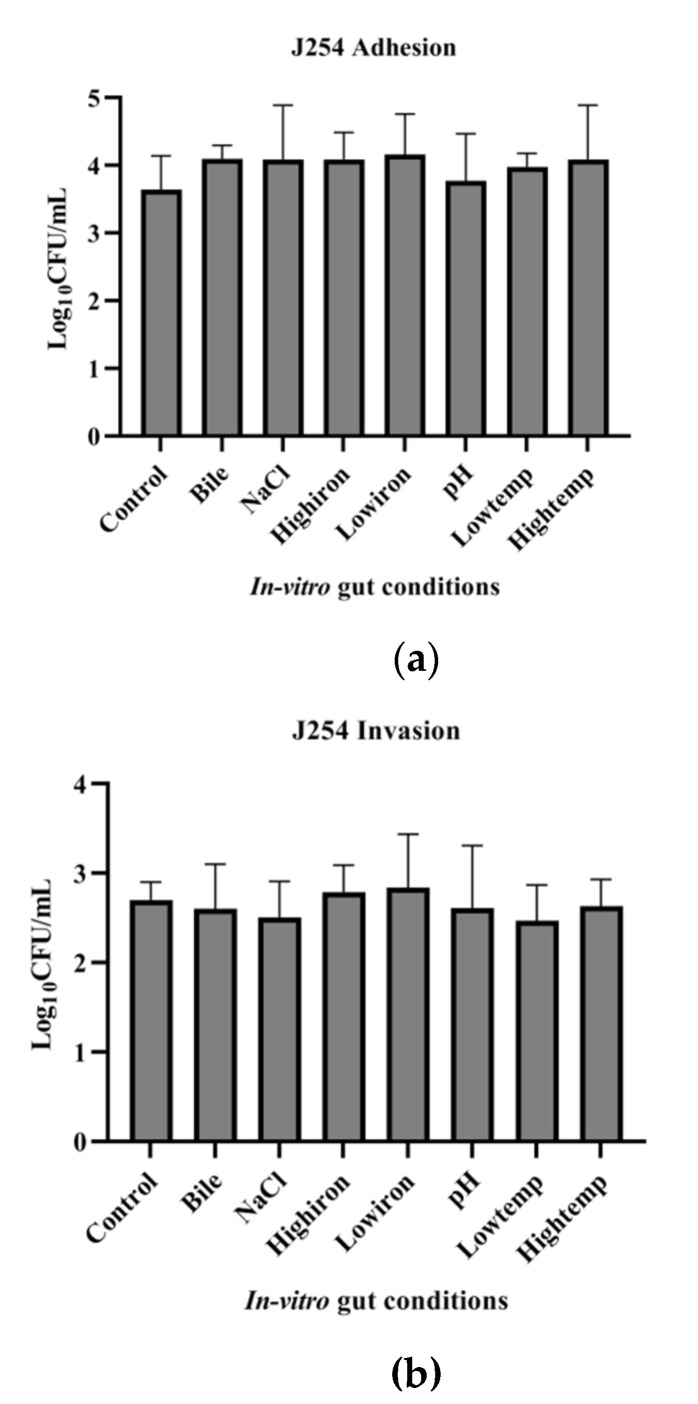
Effect of in vitro gut conditions on adhesion and invasion ability of sensitive isolate J254 to Caco-2 cell lines. Error bars represent standard deviations from triplicates. Unpaired *t*-test was performed and *p* < 0.05 was considered significant. (**a**) Effect of in vitro gut conditions on adhesion ability of sensitive isolate (J254). (**b**) Effect of in vitro gut conditions on invasion ability of sensitive isolate (J254).

The adhesion and invasion ability of J51 (MDR) and J254 (sensitive) on exposure to in vitro gut conditions were studied. Multi-drug-resistant isolate J51 showed higher adhesion ability on exposure to all the infection-related in vitro gut conditions used in the study. However, the invading ability of the isolate was lower when exposed to conditions, such as LI, bile, HT and NaCl. Under HI and pH, though not significant, the invasion ability was slightly higher than the control condition. The adhesion ability of J254 was found to be similar to that of J51. However, MDR (J51) strain showed significantly (*p* < 0.05) higher invasion ability than that of the sensitive isolate (J254). The sensitive isolate J254 showed better adhesion ability, as compared to control on exposure to HI and pH, while it showed lower invasion ability on exposure to NaCl and LT, as compared to control (Figure 7 and Figure 8).

### 3.8. Functional Classification of Differentially Expressed Genes (DEGs) in MDR Isolate on Exposure to Bile and Combination of Bile and Ciprofloaxcin 

Since the bile condition showed a significant effect on the growth and expression of virulence genes, the resistant isolate was further selected for transcriptome analysis under the bile and BAB condition. The DEGs were classified into three groups *viz* biological processes, molecular functions and cellular components by using gene ontology (Tables S4–S12). The 452 DEGs at log2 (FC)≥1 and *p*-value ≤ 0.05 of bile condition were classified into 11 functional groups of the biological process category (Figures S1 and S2), while 585 DEGs for BAB were classified into 12 functional groups of the biological process category. However, 4 functional groups were obtained out of 48 DEGs, when compared between B vs. BAB. Under the bile condition, the highest percent of DEGs belongs to sugar transport (13%) and electron transport chain (12%), while for BAB, the majority of the DEGs were under the category of ion transport and electron transport chain (17%). When compared between B vs. BAB, the majority of the DEGs were related to the tricarboxylic acid cycle (27%) and cellular response to DNA damage stimulus (18%) (Figure S3).

#### 3.8.1. Identification of Significantly Expressed Genes Using Hierarchical Clustering

The functional annotation clustering of DEGs was carried out by using DAVID. Based on the enrichment score (ES), a total of 54 clusters were produced from 452 DEGs for bile condition and the most enriched cluster were composed of genes involved in transposition and DNA recombination (ES = 3.27), and for the BAB, a total of 71 gene clusters were obtained, and the most enriched cluster (ES = 3.9) was composed of genes involved in the biosynthesis of secondary metabolism, carbon metabolism, pyruvate metabolism and citric acid cycle. For B vs. BAB, a total of 12 clusters were formed with the most enriched cluster (ES = 2.8) being genes related to metabolic pathways (Table 1).

#### 3.8.2. Downregulation of Various Metabolic Pathways on Exposure to Bile and Combination of Bile and Ciprofloxacin

KEGG pathways were obtained by using DAVID. The KEGG pathways that were downregulated by the exposure of 3% bile includes: Flagellar assembly, bacterial chemotaxis, pyruvate metabolism, starch and sucrose metabolism, biofilm formation and glycerophospholipid metabolism, while the pathway related to purine metabolism was upregulated. Upon antibiotic shock under bile condition, the biosynthesis pathways of amino acids, nucleotide sugars and secondary metabolites were downregulated. Among the metabolic pathways that were affected are pyruvate, propanoate, glutathione, glyoxylate and dicarboxylate, cysteine and methionine, butanoate, starch and sucrose, glycine, serine and threonine. Pathways that are related to cellular respiration, such as glycolysis/gluconeogenesis, citric acid cycle and oxidative phosphorylation were also downregulated. Further pathways related to flagellar and bacterial chemotaxis were also affected. However, the arginine and proline metabolism were the only upregulated pathways upon antibiotic shock under bile condition. When bile condition was compared with BAB, the genes involved in pathways related citric acid cycle, biosynthesis of secondary metabolites and amino acids were downregulated.

### 3.9. Transcriptome Validation of Differentially Expressed Genes by qPCR

The two genes that were upregulated (*csgB* and *marB*) and downregulated (*fimA* and *fliC*) in the transcriptomic analysis were selected and qPCR was performed. The obtained qPCR results were in line with the RNA sequencing data.

#### Decreased Motility on Exposure to Bile and Combination of Bile and Ciprofloxacin 

Motility was observed visually by diffused growth spreading from the line of inoculation. After overnight incubation of three tubes at 37 °C, J51 (Control) showed diffused growth throughout the medium, J51 (B) showed less diffusion in an irregular manner than J51 (Control), while the J51 (BAB) showed the least diffusion when compared to J51 (B) and control. The obtained results indicate that bile and combination of bile and antibiotic treatment resulted in reduced motility (Figure S4).

## 4. Discussion

Antibiotic resistance in human pathogens is a serious threat to public health. In this study, attempts were made to understand the effect of in vitro gut conditions on the growth kinetics, virulence gene expression, MIC variation, adhesion and invasion ability of MDR and sensitive *E. coli* isolates with or without the exposure to ciprofloxacin. Ciprofloxacin is a widely used drug for the treatment of infections caused by gut pathogens. However, increased resistance to these drugs has resulted therapeutic failures during severe infections. In the present study, among the 80 isolates, *traT* gene encoding for serum resistance has been the most frequently detected virulence factors. This is in line with the study of Rezatofighi et al. [23], wherein the majority of the isolates harboured *traT* gene. The plasmids of different size can co-exist within the same host. Plasmid profiling pattern obtained in this study did not offer much to the resistant nature of the isolates. However, the majority (80% (64)) of the MDR isolates carried at least single or multiple plasmids. This is in line with the study of Uma et al. [24], wherein the high frequency of resistance was observed among *E. coli* strains that harbored more plasmids than those without plasmids. The isolates that harbored more than three plasmids were found to be resistant to seven or more antibiotics used in the study. Nevertheless, few isolates (19% (15)) resistant to three to eight antibiotics did not carry any plasmids. This suggests that there can be a strong association of chromosomal-mediated multiple drug resistance among these isolates. The plasmids harbored by PMQR-positive isolates were found to be non-transferable even when they are subjected to in vitro gut conditions. This might be due to the absence of *traA* gene encoding for propilin, (a precursor of the pilus subunit that forms conjugative pili) in all the tested PMQR-positive isolates. 

The two isolates (J51 and J254) subjected to growth kinetics under in vitro gut conditions did not show any significant variation in growth. However, the bile, was not completely bactericidal, it showed significant effect on the growth pattern of the isolates. This is in agreement with the study of Kotian et al. [25] wherein significant reduction in the growth was seen in non-typhoidal *Salmonella* strains on exposure to bile. In addition to the effect on growth, bile also had a significant effect on the expression of virulence genes wherein all the tested genes showed downregulation in both the isolates. This is similar to the earlier reports wherein motility, flagella and invasion genes showed reduced expression on exposure to bile [26,27]. Studies have shown that iron enhances the growth, biofilm formation and virulence of *K. pneumoniae* [28,29]. However, in our study, the addition of FeCl_3_ showed a slightly enhanced growth and upregulation of virulence genes only in resistant isolate. In a study conducted by Fekri et al. [30] iron chelators prevented the growth of *E. coli* by preventing iron absorption, while in our study, the addition of iron chelator (2’2’-di-pyridyl) resulted in the extension of lag phase in sensitive isolate and slight reduction in growth in the early log phase of resistant isolate. In sensitive isolates, all the virulence genes were downregulated, but in resistant isolates, all the tested genes were upregulated. The variation in growth between the resistant and sensitive isolates, in addition to iron chelator, could be due to the absence of *iutA* gene in sensitive isolates that would otherwise facilitate the iron uptake through the expression of siderophores. Nevertheless, a large number of resistant and sensitive isolates need to be analysed to confirm these findings. Under osmotic stress, the resistant isolate showed extended lag phase, while sensitive isolates showed reduced growth in early lag phase. These results are in line with the study of Li et al. [31], wherein *E. coli* showed a decreased growth rate with the increase in osmotic stress. Further, a study conducted by Withman et al. [32] shown decreased expression of toxin and flagellar genes in UPEC with the increase in osmotic stress. This corroborates with our study, wherein the majority of the tested genes were downregulated. The acidic pH (5.8) condition did not show any significant difference in growth between two isolates, which is similar to the study of Keerthirathne et al. [33], wherein exposure to acidic pH had less effect on the growth of *Salmonella.* When bacteria are exposed to altered temperatures, the growth rate was lower in both the isolates at low temperature, while high temperatures did not have any effect on the growth pattern. However, findings of Noor et al. [34] showed normal growth of *E. coli* grown at 25 °C after 24 h exposure, while no growth was observed at 45 °C. Nevertheless, the variations in the high temperature effect could be due to the difference in the temperature (39 °C HT) in our study.

Though the in vitro gut conditions did not have any significant effect on the MIC levels for the tested antibiotics, the slight increase in MIC level for nitrofurantoin in J51 for HI, bile and for ciprofloxacin and nitrofurantoin in J254 for HI, bile and LT is the indication of isolates slowly gaining tolerance to the antibiotics under in vitro gut conditions. This gradual increase in the MIC suggests that longer exposure of sensitive isolates to these conditions, particularly bile and HI, might stimulate the isolates to become resistant to antibiotics. 

There are limited studies on the effect of ciprofloxacin on *E. coli* under in vitro gut conditions. However, the effect of sub-MIC level of ciprofloxacin on the virulence gene expression of *E. coli* was widely studied. For instance, reduced expression of iron acquisition genes, fimbriae genes and hemolysin A gene was observed on exposure to sub-MIC level of ciprofloxacin [3,35]. It was also found that the sub lethal concentration of antibiotics, such as ceftriaxone, can modulate the expression of OMPs in *S*. Typhi [13]. Similarly, in our study, the expression of tested virulence genes was found to be variable on exposure to ciprofloxacin under different in vitro gut conditions. This may be due to variation in the virulence potential of the isolates.

Both the isolates have shown varied adhesion and invasion ability in Caco-2 cells on exposure to in vitro gut conditions. On exposure to bile, both the isolates showed less invasion ability in Caco-2 cells. This is in contrast with the study of Pope et al. [36], wherein presence of bile salts enhanced the invasion ability of *shigella* spp. in HeLa cells. A study conducted by Kortman et al. [37] has shown that iron availability leads to the increased adhesion and invasion of *S.* Typhimurium to epithelial cells, which is in line with our study, wherein both the isolates showed better adhesion and invasion in Caco-2 on exposure to high iron. However, sensitive isolates showed higher invasion, when compared to resistant isolates on exposure to low iron. This might be related to the fitness of resistant isolates under low iron, wherein a large number of genes are over expressed to survive under stressful environments [38]. Further, Yoon et al. [6] have shown that exposure to NaCl increased the invasion ability of *Salmonella* Enteridis NCCP12243 in Caco-2 cells. However, in our study both the isolates showed higher adhesion but reduced invasion, when compared to control.

The RNA-seq analysis revealed the downregulation of a large proportion of genes (*n* = 173) related to metabolism pathways on antibiotic challenge under bile conditions, while 51 genes were downregulated under the bile condition only. However, earlier reports have shown an increased transcription of genes related to metabolic pathways on exposure to low concentration of ciprofloxacin. This difference might be due to the combined effect of antibiotic and bile conditions in our study [9]. Studies have shown that bile causes DNA damage in *E. coli* and *Salmonella enterica* and induces SOS responses [39]. However, only two genes (*uvrC*, *ruvA)* out of more than 50 SOS response genes were upregulated in our study. In addition, the widely used DNA damage indicator gene *dinD* showed downregulation which further indicates minimal DNA damage and higher tolerance capacity of resistant *E. coli* isolate.

**Figure 9:**
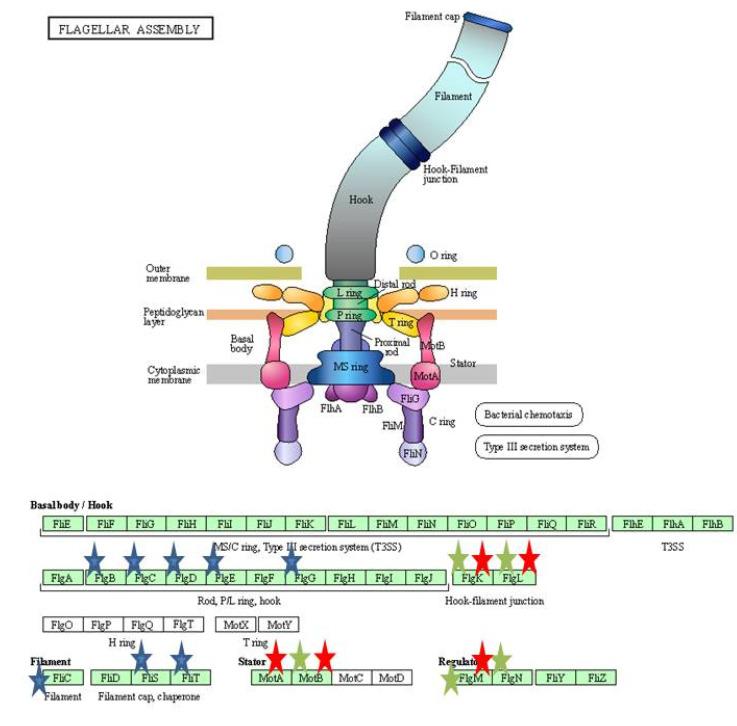
Flagellar pathway assembly (KEGG database). Blue stars represent the downregulated genes in bile and bile antibiotic treatment, green stars represent the genes downregulated in only bile treatment. Red stars represent the upregulated genes (B vs. BAB).

**Figure 10:**
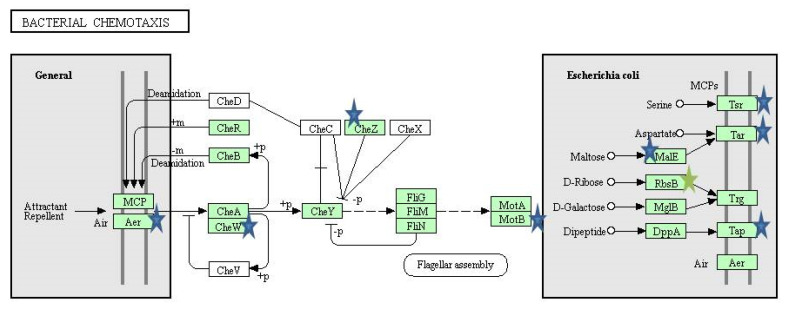
Chemotaxis pathway (KEGG database). Blue stars represent downregulated genes in bile and bile antibiotic treatment, green stars represent the genes downregulated in only bile antibiotic treatment.

Resistance to bile in bacteria is mediated by efflux pumps (AcrAB, EmrAB, and MdtABCD) or porins (*OmpF* and *ompC)*. Bile acid also induces oxidative stress genes (*micF* and *osmY*). Further, *E. coli* uses the toxin and antitoxin system (TA) MqsR/MqsA to survive at high concentration of bile [40]. The TA systems are also responsible for programmed cell death (PCD) in response to stress [41]. However, in our study the efflux pump genes, oxidative stress genes or porins were not affected while TA system gene MqsR-MqsA and other antitoxin system genes were downregulated. The repression of antitoxin system might be a reason for the *E. coli* isolate to survive in the presence of bile. Nevertheless, further studies are needed to confirm these findings. The middle flagellar genes encoding hook-basal body structures were found to be downregulated along with late flagellar genes, flagellar filament genes, motor stator and co-regulated chemotaxis genes (Figure 9 and Figure 10).

However, previous microarray results on exposure to 0.8% bile showed upregulation of middle flagellar genes and downregulation of late flagellar genes [11]. Bacteria in response to harmful conditions decrease the expression of motility genes and it could aid in self-protection through energy conservation [42]. In our study, upon antibiotic challenge all the flagellar genes were downregulated and it is in line with the previous report wherein sub-MIC or clinical levels of ciprofloxacin showed downregulation of flagellar genes [12]. 

**Figure 11:**
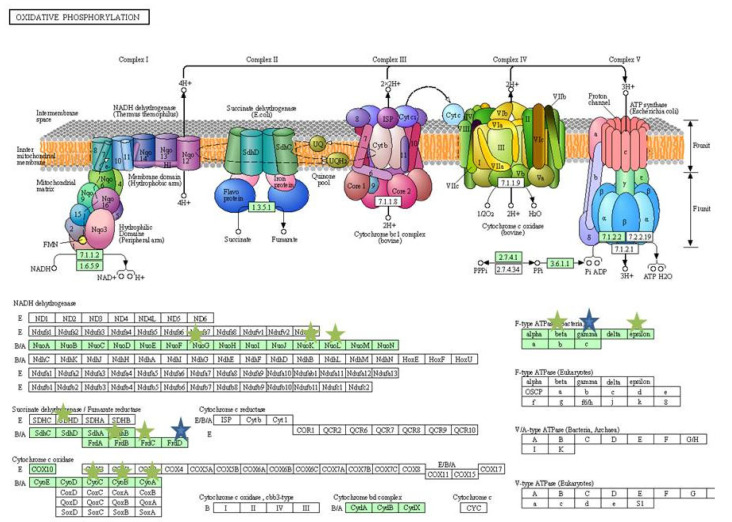
Oxidative phosphorylation (KEGG database). Blue stars represent the downregulated genes in bile and bile antibiotic treatment, green stars represent the genes downregulated in only bile antibiotic treatment.

Adhesion is the first step in initiating the infections. In this study several adhesion genes were upregulated in both the conditions. The curli formation by the extracellular components of *E. coli* is important for biofilm formation, adhesion and invasion of host cell [43]. The *CsgB*, a minor subunit of curli is required for filamentous growth in *E. coli* was found to be upregulated in both the conditions. The *ompA* gene was found to be unaffected on antibiotic challenge but was downregulated by three folds under only bile condition [11]. However, in earlier studies, exposure to bile showed upregulation of *ompA* gene. The highest level of DEGs on antibiotic challenge was found for *tisB*, encoding for Lex-A regulated proteins (log Fc = 2.51) and similar results were seen in the SOS response, in a study conducted by Valat et al. [12]. The increased expression of *tisB* resulted in decreased proton motive force and ATP levels (Figure 11), which are in agreement with the study of Dorr et al. [44,45,46].

## 5. Conclusions

Understanding the adaptive ability and virulence potential of the bacteria to the host physiological conditions and unravelling the genetic cross talk between the virulence and resistance nature of bacteria helps in combating infections caused by them. Many of the gut physiological conditions that were earlier thought to be antibacterial are now found to influence the growth and survival of the bacteria. In addition, the possible conversion of susceptible isolates to resistant or vice versa, due to the impact of gut conditions during infection, cannot be ruled out. In the present study, the resistant isolate was able to withstand the exposed conditions better than the sensitive isolate. However, differential expression of virulence genes was observed between sensitive and resistant isolates on exposure to in vitro gut conditions and antibiotic challenge. This suggests that gut physiological conditions and antibiotics have a profound effect on the survival and virulence of drug resistant *E. coli*. The in vitro gut conditions and antibiotic challenge increased the fitness of resistant isolate. This further suggests that the use of antibiotics during infection could enhance the virulence potential of multidrug-resistant *E. coli*, thereby leading to therapeutic failure. Considering the impact of in vitro gut conditions on the drug-susceptible and resistant pathogens, it might be necessary to revise the treatment regimes while treating the notorious human pathogens in the near future to combat AMR. This was a first-of-its-kind study that showed the effect of antibiotics and in vitro gut conditions on drug-resistant and sensitive isolates. This study could pave the way to understand the variable nature of drug-resistant and sensitive *E. coli* during infection in vivo. Nevertheless, many drug-resistant and sensitive isolates need to be tested to support these findings.

## Figures and Tables

**Table 1:** Summary of functional annotation clustering analysis by DAVID tools. Clusters ES threshold was set to 2 and gene count ≥4 was only represented in table.

Functional Annotation Cluster	Enrichment Score	Gene Count
**C vs. B**		
**Annotation Cluster 1**	3.27	
Transposition		10
DNA Recombination		16
**Annotation Cluster 2**	2.71	
Bacterial type flagellum-dependent cell motility		11
Bacterial type flagellum dependent swarming facility		8
**Annotation Cluster 3**	2.31	
Flagellar assembly		14
**C vs. BAB**		
Annotation Cluster 1	3.9	
Biosynthesis of secondary metabolites		62
Carbon metabolism		31
Pyruvate metabolism		19
Citric acid cycle		12
**Annotation Cluster 2**	3.55	
DNA recombination		19
Transposition		15
**Annotation Cluster 3**	2.82	
Glycolysis/Gluconeogenesis		15
**Annotation Cluster 4**	2.6	
NADP binding		20
**B vs. BAB**		
**Annotation Cluster 1**	2.8	
Metabolic pathways		12
**Annotation Cluster 2**	2.17	
ATP binding		10
Response to heat		6

## Data Availability

The data presented in this study are available as supplementary files. Figure S1–S4, Tables S1–S12.

## Supplementary Materials

The following supporting information can be downloaded at: https://www.mdpi.com/article/10.3390/ijerph191912805/s1, Figure S1: A—Gene ontology distribution under various biological processes that are upregulated between C vs. B. B—Gene ontology distribution under various biological processes that are down regu-lated between C vs. B. C—Gene ontology distribution under various biological processes that are upregulated between B vs. BAB. D—Gene ontology distribution under various biological pro-cesses that are upregulated between B vs. BAB, Figure S2: E—Gene ontology distribution under various biological processes that are upregulated between BT vs. BAB. F—Gene ontology dis-tribution under various biological processes that are down regulated between BT vs. BAB, Figure S3: A. Gene ontology of DEGs after exposure to bile B. Gene ontology of DEGs after exposure to bile + antibiotic shock C. Gene ontology of DEGs compared between bile and bile + antibiotic shock, Figure S4: Validation of transcriptome data by motility test using stab method. A—J51 (Control) showed diffused growth throughout the medium, B—J51 (B) bile treated showed less diffusion in an irregular manner, C—J51 (BAB) bile + antibiotic showed least diffusion through-out the medium, Table S1: Details of primers used in the study for the amplification of Different genes, Table S2: MIC variation under control and in vitro gut conditions for isolate J51, Table S3: MIC variation under control and in vitro gut conditions for J254, Table S4: Flagellar genes that were differentially expressed upon bile (B), bile and antibiotic (BAB) treatment, Table S5: Adhesion genes that were differentially expressed upon bile (B), bile and antibiotic (BAB) treatment, Table S6: Sugar catabolism and TCA cycle genes that were differentially expressed upon bile (B), bile and antibiotic (BAB) treatment, Table S7: Proton transport and electron transport chain genes that were differentially expressed upon bile (B), bile and antibiotic (BAB) treatment, Table S8: Amino acid catabolism genes that were differentially expressed upon bile (B), bile and anti-biotic (BAB) treatment, Table S9: Envolope and periplasmic genes that were differentially ex-pressed upon bile (B), bile and antibiotic (BAB) treatment, Table S10: Stress response genes that were differentially expressed upon bile (B), bile and antibiotic (BAB) treatment, Table S11: Lipo-proteins genes that were differentially expressed upon bile (B), bile and antibiotic (BAB) treat-ment, Table S12: Antibiotic resistance genes that were differentially expressed upon bile (B), bile and antibiotic (BAB) treatment.
