# Supplementary material for: Survival and Virulence Potential of Drug-Resistant E. coli in Simulated Gut Conditions and Antibiotic Challenge
Source: Int J Environ Res Public Health. 2022 Oct 6;19(19):12805. doi: 10.3390/ijerph191912805 (PMC9566084; doi:10.3390/ijerph191912805)
Supplement: Supplementary file 1 [file ijerph-19-12805-s001.zip › ijerph-1887975-supplementary.pdf]

Table S1. Details of primers used in the study for the amplification of Different genes.

Virulence genes	Primer sequence	Size	Reference
cnf-1	F- CGTCGGAATACCTGCATTTT<br>R- ACGCTCATCAAGCTCTCCAT	162	This Study
traT	F- GAGACCATCTGGCTTGAACC<br>R- TCCCGCAGATCCATCTTATC	203	
ompT	F- ATCTAGCCGAAGAAGGAGGC<br>R- CCCGGGTCATAGTGTTCATC	174	
IbeA	F-AGTTGTTGGTGGTGGTCCAT<br>R-CCATTGATTTTGCCGTTTCT	206	
csgB	F-AATCAGGCAGCCATAATTGG<br>R-CCATAAGCACCTTGCGAAAT	200	
marB	F-CACTTTCATCCGCAATAGCA<br>R-AGCGCATCCGACTTATCACT	178	
fliC	F-AGCGCATCCGACTTATCACT<br>R-AGCGCATCCGACTTATCACT	194	
fimA	F-AGCGCATCCGACTTATCACT<br>R-AGCGCATCCGACTTATCACT	167	
rfc	F-AGGATCCATTGCCAGTGAAA<br>R-GCGAGTCATAACTGGCATCA	247	
traA	F-GATGTTCTTCCCGCAACTGG<br>R-CCGTCGTTGGCCTGAAATAA	238	
TraB	F-AATGCCTCTGTCACCGGTAA<br>R-TTTACCCCGGAAGCTGACAT	234	
FimH	F-GGTGAAAACGACGCGGTATT<br>R-GATGTCACCGTTACTCTGCC	152	[14]
PapC	F-GCCAGTGCCGTTGAGTTTAA<br>R-TCTGTAACGATGCGGGAGAA	157	
iutA	F-ACGTCTTTCCGTGGCATATC<br>R-TTAAGCCCGTAATCGTCGTC	207	
16sRNA	F- CGGACGGGTGAGTAATGTCT<br>R- CTCAGACCAGCTAGGGATCG	100	

Table S2. MIC variation under control and *in-vitro* gut conditions for isolate J51.

In-vitro gut condition	NA (mcg)	AMP (mcg)	COT (mcg)	NIT (mcg)	CIP (mcg)	C (mcg)	TE (mcg)	IMP (mcg)
Control	>256	>256	>256	8	>256	3	8	0.19
High Iron	>256	>256	>256	12	>256	3	8	0.19
Low Iron	>256	>256	>256	8	>256	3	8	0.19
Bile	>256	>256	>256	12	>256	3	8	0.19
pH	>256	>256	>256	8	>256	3	8	0.19
LT	>256	>256	>256	8	>256	3	6	0.19
HT	>256	>256	>256	8	>256	3	8	0.19
NaCl	>256	>256	>256	12	>256	3	8	0.19

Table S3. MIC variation under control and *in-vitro* gut conditions for J254.

In-vitro gut condition	NA (mcg)	AMP (mcg)	COT (mcg)	NIT (mcg)	CIP (mcg)	C (mcg)	TE (mcg)	IMP (mcg)
Control	4	>256	0.94	8	0.094	3	4	0.19
High Iron	4	>256	0.94	12	0.125	3	4	0.19
Low Iron	4	>256	0.94	8	0.064	3	4	0.19
Bile	4	>256	0.94	12	0.125	3	4	0.19
pH	4	>256	0.94	8	0.094	3	4	0.19
LT	4	>256	0.94	6	0.125	3	4	0.19
HT	4	>256	0.94	8	0.094	3	4	0.19
NaCl	4	>256	0.94	6	0.094	3	4	0.19

NA- Nalidixic acid, **AMP**- Ampicillin, **COT**- Co-trimoxazole, **NIT**- Nitrofurantoin, **CIP**- ciprofloxacin, **C**- chloramphenicol, **TE**- Tetracycline, **IMP**- Imipenem.

Table S4. Flagellar genes that were differentially expressed upon bile (B), bile and antibiotic (BAB) treatment

Gene	Function	B	BAB
cheA	Chemotaxis sensor kinase	-1.78	-
cheW	Chemotaxis signal transducer	-1.63	-1.25
cheZ	CheY-P phosphatase	-2.16	-2.09
fliS	Cytosolic chaperone inhibits premature FliC assembly	-1.88	-1.71
fliT	Flagellar synthesis	-1.53	-1.17
flgB	Basal body rod subunit	-1.90	-1.86
flgC	Basal body rod subunit	-1.86	-1.67

flgD	Basal body rod modification	-2.28	-1.96
flgE	Hook subunit	-2.01	-1.79
flgG	Basal body rod major subunit	-2.04	-1.84
flgK	Flagellar synthesis	-2.01	-
flgL	Flagellar synthesis	-2.19	-
fliA	Sigma 28; regulates class III flagellar genes	-1.84	-1.34
fliC	Flagellin subunit, H-antigen	-4.79	-4.58
motB	Flagellar rotation	-1.27	-
ycgR	Suppresses *hns* motility defect	-1.21	-
yhjH	Suppresses *hns* motility defect	-1.71	-

Table S5. Adhesion genes that were differentially expressed upon bile (B), bile and antibiotic (BAB) treatment

ompA	Outer membrane protein A porin - cell shape/integrity, adhesion	-	-3.01
ydeQ	F9 fimbriae - predicted adhesin	1.49	1.43
ydeR	F9 fimbriae - predicted adhesin	1.89	2.02
ydeS	F9 fimbriae - predicted adhesin	1.66	1.70
znuA	zinc transporter subunit: periplasmic-binding component of ABC superfamily	1.65	1.38
csgB	curlin nucleator protein, minor subunit in curli complex	1.55	1.40

Table S6. Sugar catabolism and TCA cycle genes that were differentially expressed upon bile (B), bile and antibiotic (BAB) treatment

Gene	Function	B	BAB
aceE	Pyruvate dehydrogenase	-	-2.67
aceF	Pyruvate dehydrogenase dihydrolipoamide acetyltransferase	-2.68	-2.80
acnB	Aconitase B; 2-methylaconitate hydratase	-	-2.80
dld	D-Lactate dehydrogenase	-1.21	-1.38
galK	Galactokinase	-1.06	-1.03
galM	Galactose mutarotase; aldose-1-epimerase	-1.68	-1.80
glpA	Glycerol-3-phosphate dehydrogenase large subunit (anaerobic)	-2.72	-2.70
glpB	Glycerol-3-phosphate membrane anchor (anaerobic)	-2.72	-2.48
glpC	Glycerol-3-phosphate dehydrogenase (anaerobic) small subunit	-3.10	-2.81
gnd	Gluconate-6-phosphate dehydrogenase	-	-1.53
icdA	Isocitrate dehydrogenase	-	-1.89

malE	Maltose-binding protein, periplasmic	-1.50	-1.41
malM	Periplasmic protein, *mal* regulon	-1.46	-1.31
pdhR	Pyruvate dehydrogenase operon repressor	-	-1.85
pflB	Pyruvate formate lyase I (anaerobic)	-	-3.14
pgi	Glucose phosphate isomerase	-	-1.66
pta	Phosphotransacetylase	-	-2.24
srlA	Sorbitol-specific enzyme II of PTS	-1.09	-
srlB	Sorbitol-specific enzyme III of PTS	-1.37	-1.15
srlD	Sorbitol-6-phosphate dehydrogenase	-1.64	-1.47
srlE	srl operon protein	-1.23	-
tpiA	Triosephosphate isomerase	-	-1.94

Table S7. Proton transport and electron transport chain genes that were differentially expressed upon bile (B), bile and antibiotic (BAB) treatment

Gene	Function	B	BAB
atpC	ATP synthase subunit epsilon, F1	-2.22	-3.10
atpD	ATP synthase subunit beta, F1	-	-2.49
atpG	ATP synthase subunit gamma, F1	-	-2.24
cyoA	Cytochrome *o* oxidase subunit II	-	-1.94
cyoB	Cytochrome *o* oxidase subunit I	-	-1.62
cyoC	Cytochrome *o* oxidase subunit III	-	-1.34
frdC	Fumarate reductase membrane anchor polypeptide	-	-2.64
ndh	Respiratory NADH dehydrogenase II; NADH:ubiquinone oxidoreductase II	-1.21	-1.33
nuoG	NADH:ubiquinone oxidoreductase subunit G; NADH dehydrogenase I	-	-1.44
nuoK	NADH:ubiquinone oxidoreductase subunit K; NADH dehydrogenase I	-	-1.39
nuoL	NADH:ubiquinone oxidoreductase subunit L; NADH dehydrogenase I	-	-1.30
sdhD	Succinate dehydrogenase hydrophobic subunit	-	-1.22

Table S8. Amino acid catabolism genes that were differentially expressed upon bile (B), bile and antibiotic (BAB) treatment

Gene	Function	B	BAB
artM	Arginine periplasmic binding protein	1.57	-
cysK	*O*-Acetylserine sulfhydrylase A (cysteine synthase)	-	-1.98
lysU	Lysine-tRNA ligase	-	-1.23
tnaA	Tryptophan deaminase, degradative; also deaminases serine and cysteine	1.64	1.37

tnaC	*tnaA* leader peptide	1.19	-
ydfG	L-*allo*-Threonine, L-serine, D-serine dehydrogenase	-	-1.22

Table S9. Envelope and periplasmic genes that were differentially expressed upon bile (B), bile and antibiotic (BAB) treatment

Gene	Function	B	BAB
fadL	Fatty acid transport, outer membrane	-1.10	-1.48
hlpA	Periplasmic chaperone for OMPs	-	-2.37
lamB	Maltoporin, maltose high-affinity uptake; phage lambda receptor	-1.74	-1.49
malE	Maltose-binding protein, periplasmic	-1.50	-1.41
malM	Maltose operon periplasmic protein	-1.46	-1.31
ompT	Outer membrane protease VII	1.03	1.13
rbsB	D-Ribose binding protein, periplasmic	-	-1.36
tpX	Thiol peroxidase, antioxidant	-	-2.32
fimA	Major type 1 subunit fimbrin (pilin)	-1.97	-2.51
ompA	Outer membrane protein 3a	-	-3.01

Table S10. Stress response genes that were differentially expressed upon bile (B), bile and antibiotic (BAB) treatment

Gene	Function	B	BAB
ahpC	Alkyl hydroperoxide reductase	-	-2.32
ahpF	NAD(P)H:peroxiredoxin oxidoreductase	-1.81	-2.20
cysK	Cysteine synthase, *o*-acetylserine sulfhydrylase A	-	-1.98
dnaK	HSP-70-type molecular chaperone	2.25	-
dnaJ	DnaK cochaperone	2.07	-
dinJ	Induced by DNA damage	-1.92	-
hslV	Heat-inducible ATP-dependent protease	-1.97	-
katG	Catalase-hydrogen peroxidase I	-3.00	-3.15
uspD	UV resistance	-1.94	-2.26
cspA	RNA chaperone and anti-terminator, cold-inducible	2.76	-
rmf	ribosome modulation factor	2.65	-
safA	two-component system connector membrane protein, EvgSA to PhoQP	1.67	1.41
yoeI	Un charecterized protein	1.77	-
ycgZ	RcsB connector protein for regulation of biofilm and acid-resistance	-	1.13
cspA	RNA chaperone and anti-terminator, cold-inducible	2.76	-

cspG	Cold shock protein homolog, cold-inducible	1.21	2.02
uspA	Universal stress global response regulator	-2.96	-3.59

Table S11. Lipoproteins genes that were differentially expressed upon bile (B), bile and antibiotic (BAB) treatment

Gene	Function	B	BAB
metQ	DL-methionine transporter subunit	-1.12	-1.02
yeaY	Slp family lipoprotein, RpoE-regulated	-	1.28
ybjP	lipoprotein	-2.02	-2.30
ybaY	outer membrane lipoprotein	-1.80	-1.72
osmE	osmotically-inducible lipoprotein	-2.11	-2.37
pal	peptidoglycan-associated outer membrane lipoprotein	-	-2.17
slp	Slp outer membrane lipoprotein	-	-1.92

Table S12. Antibiotic resistance genes that were differentially expressed upon bile (B), bile and antibiotic (BAB) treatment

Gene	Function	B	BAB
relE	Qin prophage; toxin of the RelE-RelB toxin-antitoxin system	-1.08	-
blr	beta-lactam resistance membrane protein; divisome-associated protein	1.03	-
mdtJ	multidrug efflux system transporter	1.07	-
marB	multidrug efflux system transporter	1.34	1.47
tehA	potassium-tellurite ethidium and proflavin transporter	1.16	1.44
ydhC	putative arabinose efflux transporter	2.83	2.20
mgrR	ncRNA	1.84	1.75
rlmN	dual specificity 23S rRNA m(2)A2503, tRNA m(2)A37 methyltransferase, SAM-dependent	1.06	-
sapB	antimicrobial peptide transport ABC transporter permease	1.03	1.09
emrE	DLP12 prophage; multidrug resistance protein	-	1.39
ychE	UPF0056 family inner membrane protein	1.02	1.04
glmS	L-glutamine:D-fructose-6-phosphate aminotransferase	-2.01	-2.41
arsB	arsenite/antimonite transporter	-1.29	-1.20
glgP	glycogen phosphorylase	-1.22	-1.41
malP	maltodextrin phosphorylase	-1.35	-1.38
yeaR	DUF1971 family protein, nitrate-inducible	-	1.11
mdtL	multidrug efflux system transporter	-	1.08
mdtK	multidrug efflux system transporter	-	1.30

tolC	transport channel	-	-1.81

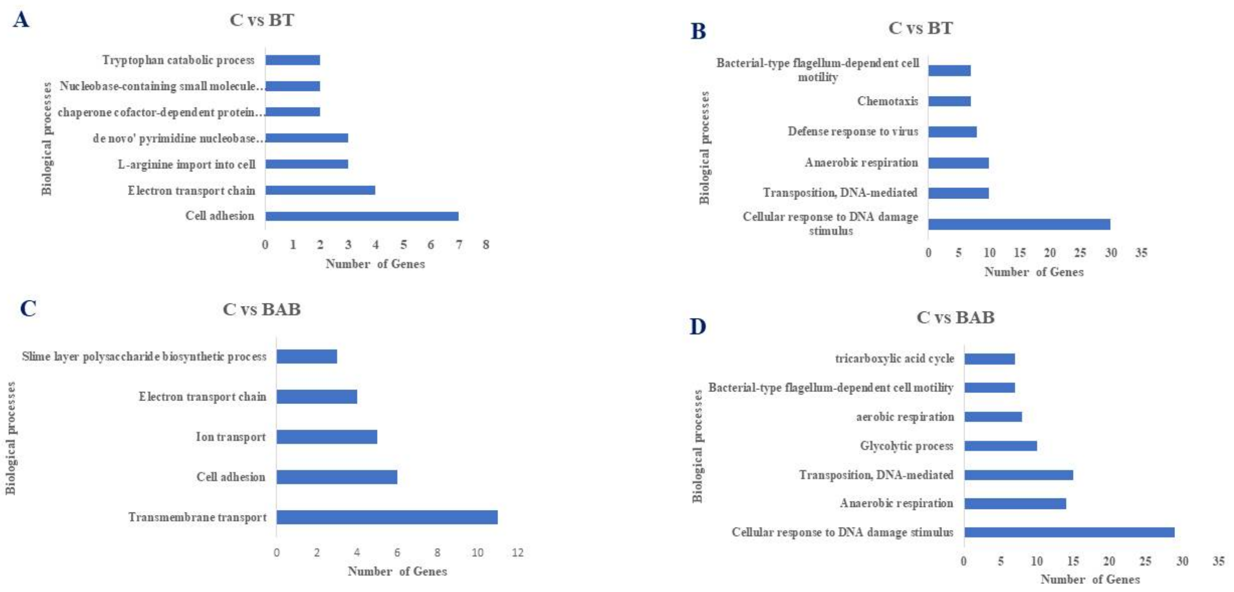

Figure S1. **A** - Gene ontology distribution under various biological processes that are upregulated between C vs B. **B** - Gene ontology distribution under various biological processes that are down regulated between C vs B. **C** - Gene ontology distribution under various biological processes that are upregulated between B vs BAB. **D** - Gene ontology distribution under various biological processes that are upregulated between B vs BAB.

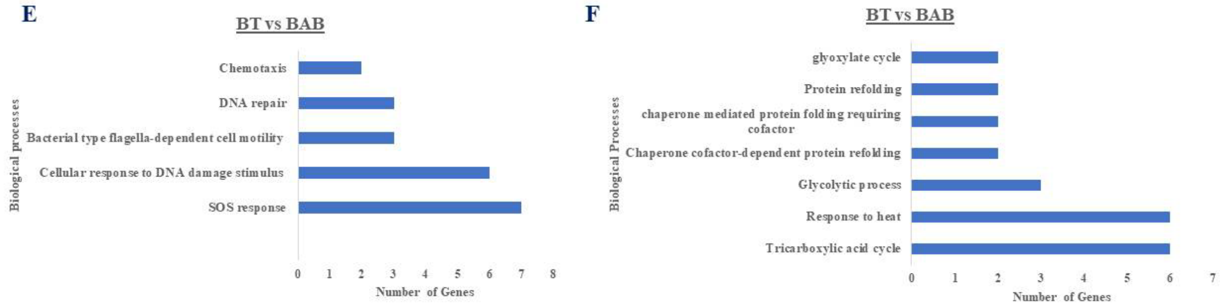

Figure S2. **E**- Gene ontology distribution under various biological processes that are upregulated between BT vs BAB. **F**- Gene ontology distribution under various biological processes that are down regulated between BT vs BAB.

Functional categorization of DEGs based on biological processes (C vs B)

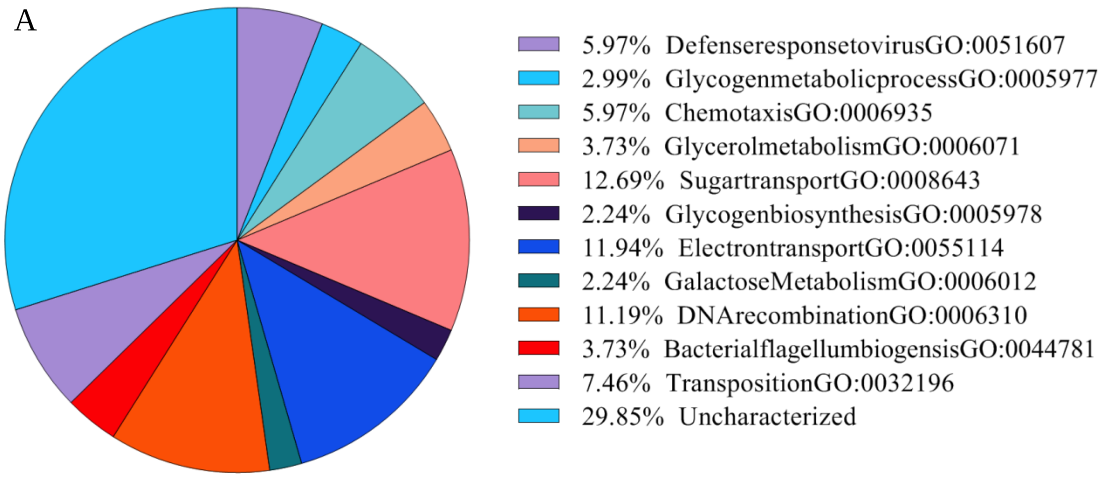

Functional categorization of DEGs based on biological processes (C vs BAB)

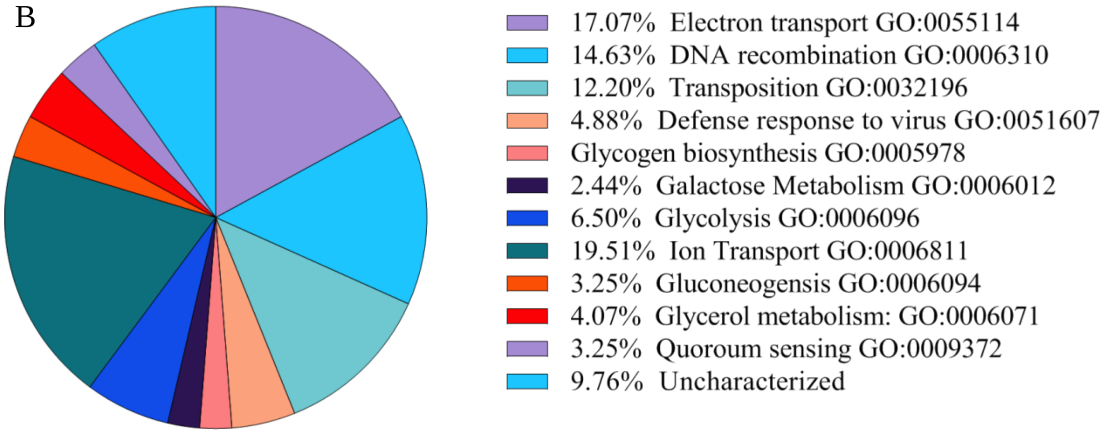

Functional categorization of DEGs based on biological processes (B vs BAB)

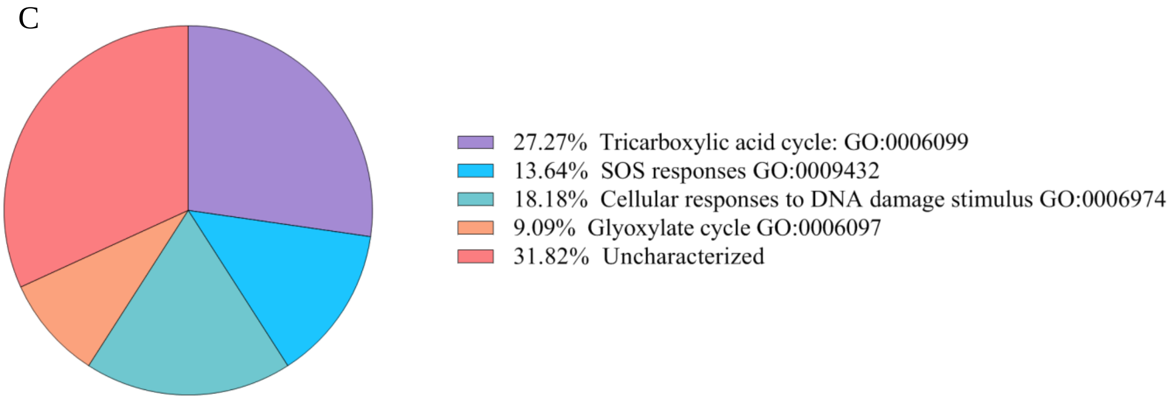

Figure S3. **A.** Gene ontology of DEGs after exposure to bile **B.** Gene ontology of DEGs after exposure to bile + antibiotic shock **C.** Gene ontology of DEGs compared between bile and bile + antibiotic shock.

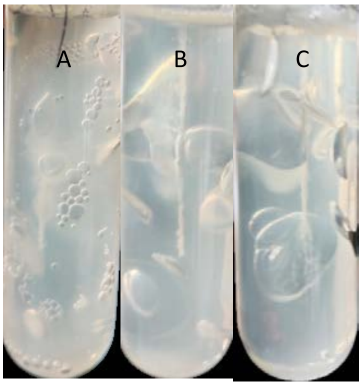

Figure S4. Validation of transcriptome data by motility test using stab method. A- J51 (Control) showed diffused growth throughout the medium, B- J51 (B) bile treated showed less diffusion in an irregular manner, C- J51 (BAB) bile + antibiotic showed least diffusion throughout the medium.
